# Acridine Orange Indicates Early Oxidation of Wood Cell Walls by Fungi

**DOI:** 10.1371/journal.pone.0159715

**Published:** 2016-07-25

**Authors:** Carl J. Houtman, Peter Kitin, Jon C. D. Houtman, Kenneth E. Hammel, Christopher G. Hunt

**Affiliations:** 1 USDA Forest Service, Forest Products Laboratory, One Gifford Pinchot Drive, Madison, Wisconsin, United States of America; 2 Department of Bacteriology, University of Wisconsin, Madison, Wisconsin, United States of America; 3 Department of Microbiology, University of Iowa, Iowa City, Iowa, United States of America; INRA, FRANCE

## Abstract

Colonization of wood blocks by brown and white rot fungi rapidly resulted in detectable wood oxidation, as shown by a reduced phloroglucinol response, a loss of autofluorescence, and acridine orange (AO) staining. This last approach is shown to provide a novel method for identifying wood oxidation. When lignin was mildly oxidized, the association between AO and lignin was reduced such that stained wood sections emitted less green light during fluorescence microscopy. This change was detectable after less than a week, an interval that past work has shown to be too short for significant delignification of wood. Although fungal hyphae were observed in only a few wood lumina, oxidation was widespread, appearing relatively uniform over regions several hundred micrometers from the hyphae. This observation suggests that both classes of fungi release low molecular weight mild oxidants during the first few days of colonization.

## Introduction

Determination of the spatial and temporal distribution of ligninolytic agents during fungal colonization of wood has been the focus of many studies. Schwarze [1] comprehensively reviewed methods for microscopically characterizing wood decay. For example, Srebotnik and Messner [2] found that by staining decayed wood sections with astra-blue and safranin they were able to observe regions where enzyme action by white rot fungi had exposed cellulose surfaces.

With lignin’s lack of hydrolyzable linkages, oxidation is likely the only route to lignin biodegradation [3]. Recent work indicates that enzymes and low molecular weight metabolites with a role in lignin oxidation are found across diverse fungal families [4, 5]. In addition, results from cultures of both white rot and brown rot fungi grown on wood show that lignin is oxidatively cleaved during decay [6–8]. Moreover, recent observations using an immobilized oxidation-sensitive dye indicate that gradients in the extent of oxidation occur around the hyphae of the most intensively studied white rot fungus, *Phanerochaete chrysosporium* [9]. However, it remains uncertain which oxidants initiate ligninolysis [10], in part because current methods for detection of oxidation in wood yield useful data only after extensive decay, at which time multiple agents have likely contributed. Our goal here was to find an improved, convenient method to detect onset of oxidation during incipient wood decay.

If detection of oxidation at an early stage of decay is the objective, staining with phloroglucinol [11] could provide an approach. Under acid conditions, phloroglucinol reacts with aryl aldehydes to yield a red compound [12]. Because coniferyl and sinapyl aldehydes naturally exist in wood lignin and are easily oxidized, they are likely depleted in oxidized wood, and thus a reduced phloroglucinol response might serve as an early indicator of fungal attack. However, because the observations are made with transmitted light microscopy, many instrument factors can change the apparent intensity of the red color, and thus this method is difficult to use quantitatively.

As an alternative, we have refined a test method based on staining with acridine orange (AO) and observation by fluorescence microscopy. Our approach is able to detect modification of wood biopolymers within days of exposure to a fungus as a change in color. Previous application of this test method [13] was limited to observations of extensive generalized decay, as opposed to detection of incipient biodegradation. The improved AO staining method has the potential to facilitate studies of biochemical mechanisms active during fungal biodegradation of wood.

Since the 1940s [14, 15], when AO was first proposed as a fluorescent stain, it has been used widely in microscopic applications. The most common application is to stain DNA and RNA [16, 17]. In a clinical setting, AO is widely used to screen for bacteria in blood and central nervous system fluid [18]. In a research setting, AO can differentially stain double-stranded and single-stranded nucleic acids [19]. The association of polymeric nucleic acids with AO appears to have two mechanisms. With double stranded material, the dominant mode is intercalation between the base pairs [20], which electronically isolates the AO molecules and results in green fluorescence upon UV exposure. By contrast, with single-stranded material, interactions with anionic groups on the nucleic acid dominate, which favors stacking of the cationic AO molecules [21]. Stacking results in a combined electronic state between two AO molecules, giving a red fluorescent emission and the potential for fluorescent resonance energy transfer (FRET) that quenches the green emission of any isolated AO molecules within the Förster distance.

**Fig 1:**
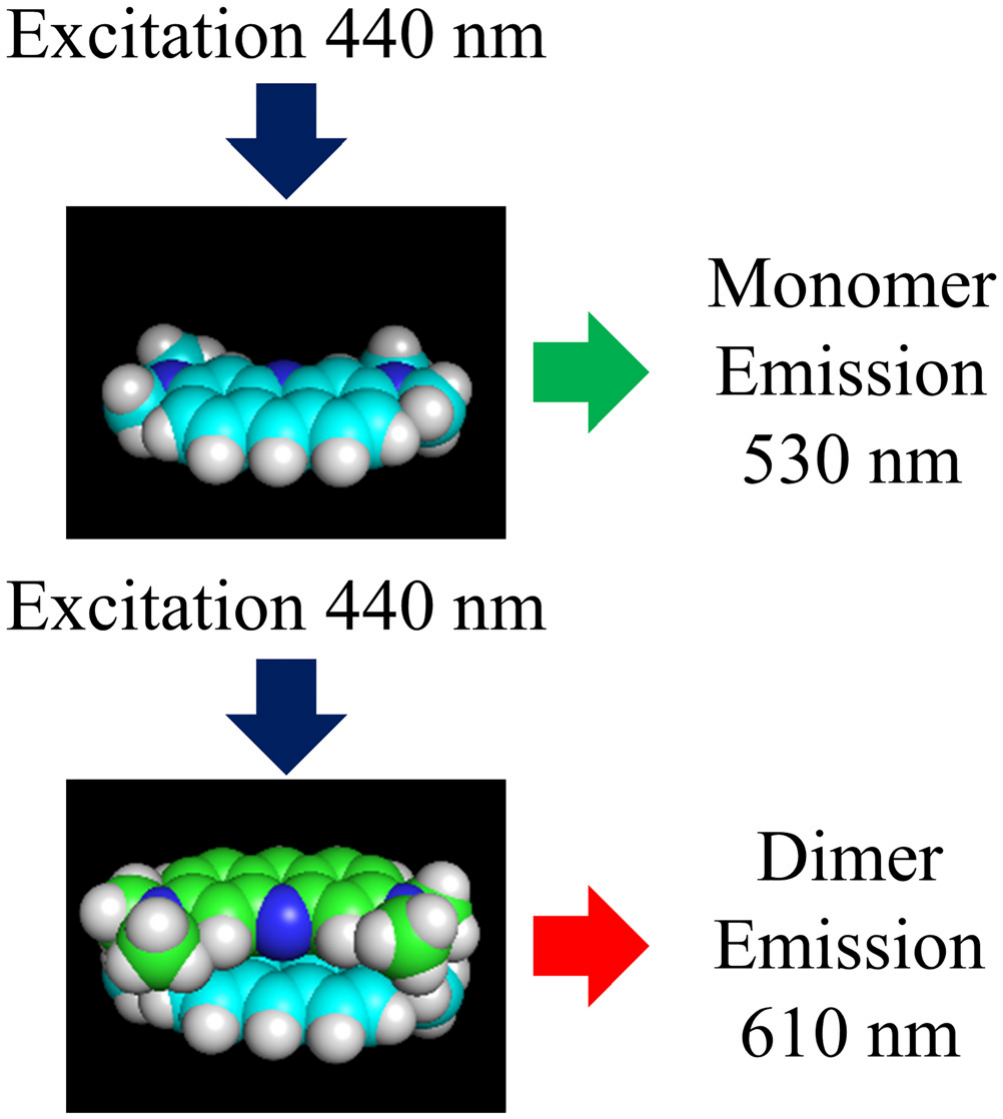
Space-filling representation of AO monomer and dimer. The representation was generated with pyMOL 1.8. The distance between the AO molecules in the dimer was set to 0.34 nm, as described by Davies *et al*. [23].

AO in water solutions forms relatively stable dimers with binding energies near 40 kJ/mol and equilibrium constants between 10^3^ and 10^4^ L/mol at room temperature [22] For an equilibrium constant of 5,000 L/mol and 1,000 μmol/L total AO concentration, 73% of the AO molecules are expected to be associated as dimers, whereas at 1 μmol/L, <1% are dimers. AO dimers in water are oriented with planes of the rings parallel and separated by 0.34 nm, but twisted by 144° [23]. Space-filling representations of the AO monomer and dimer are shown in Fig 1. The approximately opposite orientation optimizes dipolar association and accounts for steric limitations caused by methyl groups. Cundall *et al*. [24] proposed similar sandwich-like structures to explain experimental observations involving complexes of AO with various polyanions. AO has a strong propensity to adsorb on surfaces and is a sensitive probe of the binding environment presented by various substrates [25].

Some efforts were previously made to understand the staining of wood and other plant tissues by AO. Drnovseka and Perdih [26], who studied a wide range of dyes for wood fiber characterization, observed that AO has affinity for lignin. Stockert *et al*. [27] compared the staining of bean stem transverse sections with several fluorescent dyes. When they applied 1000 μmol/L solutions of AO to fixed sections and then washed with distilled water, lignified tissues produced a bright green emission. The authors proposed that associations between lignin and hydrophobic dyes results in isolated fluorescent centers. Similar results were observed by Li and Reeve [28] for pulped wood fibers. They noted that for AO concentrations below 1 μmol/L, only green emission was observed and emission intensity positively correlated with lignin content. They also noted, however, that at higher dye concentrations, the red emission characteristic of AO dimers was observed. When Liu *et al*. [29] used 100 μmol/L AO solutions to stain fibers, they found that red emission remained relatively constant for fibers of different lignin levels, which suggests that the formation of AO dimers on lignocellulose does not result from associations with lignin. It was further observed that staining of microcrystalline cellulose with AO (at <0.5 μmol/g of substrate) resulted in the green emission of isolated AO molecules [30], which indicates that cellulose is also not the location at which AO dimers associate on lignocellulose.

In a related line of research, other cationic dyes besides AO have been used to characterize and measure anionic groups on surfaces and polymers. For example, methylene blue, a cationic dye structurally similar to AO, is the basis of a method for titrating anionic groups on pulp fibers [31]. Similarly, Mathews *et al*. [32] used a dye mixture to measure the charge demand of pulp fibers. In this work, they used AO red emission as a reference for the blue emission of another dye, which indicates that AO dimers bind strongly to a small number of sites, but the monomer can be excluded from the majority of the anionic sites by the other dye.

A working hypothesis consistent with all these results is that lignin in native lignocelluose adsorbs dilute AO weakly as monomers at isolated sites, which gives green emission. At higher AO concentrations, anionic polysaccharides provide a small number of additional sites that can be saturated with AO dimers, which then exhibit their characteristic red emission. As lignin is modified by oxidation, fewer isolated sites are available for AO monomers to bind, which reduces green emission. Finally, as lignin and hemicellulose removal approaches completion, AO can bind as isolated molecules on cellulose surfaces, and green fluorescence thus reappears.

The purpose of this publication is to demonstrate the utility of AO for studying very early fungal decay and to test our working hypothesis on the mechanism of metachromicity by exposing various defined substrates to AO solutions. Specifically, we are interested in determining which components of wood cell walls are likely responsible for the color shift in staining of wood sections after oxidation.

## Experimental

### Control

Transverse and longitudinal sections 40 μm thick were cut from green white spruce sapwood blocks (1×1×4 cm) using a microtome. The 4-cm dimension was in the longitudinal direction. The blocks were stored frozen and were thawed before sectioning. To ensure that wood sample surfaces were not previously oxidized, sections were taken at approximately 2 mm below the wood surface. Sections were saved refrigerated in a covered glass Petri dish.

### Transverse sections treated with acetic acid and acid chlorite

Transverse sections were treated with acid chlorite according to a procedure similar to that of Ahlgren and Goring [33]. One hundred sections were added to a solution that contained 30 mL of water, 0.2 mL of acetic acid, and 0.005 g of sodium chlorite. This mixture was stirred constantly and held at 22°C. Acetic acid and sodium chlorite were added to the solution according to Table 1.

**Table 1:** Chemical addition schedule for low acid-chlorite treatment.

Time (min)	Acetic acid (mL)	Chlorite (g)
0	0.2	0.005
5	0.2	0.005
15	0.2	0.005
25	0	0.005
35	0	0.005

After being stirred for 55 min, the samples were rinsed five times with distilled, deionized (DI) water and placed in a covered glass Petri dish in a refrigerator. A separate group of transverse sections had the same treatment and acid addition schedule except no chlorite was added. These sections were designated as acetic acid-treated.

### Fully delignified longitudinal sections

Fully delignified longitudinal sections were prepared according to Ahlgren and Goring [33] using more severe conditions than in the procedure just outlined. Longitudinal sections from the radial face were chosen because transverse sections disintegrate upon complete delignification. Ten sections were placed in a beaker that contained 30 mL of water, 0.033 mL of acetic acid, and 0.1 g of sodium chlorite. This solution was held at a constant temperature of 70°C. Acetic acid and sodium chlorite were added to the solution according to Table 2.

**Table 2:** Chemical addition schedule for full delignification treatment.

Time (h)	Acetic acid (mL)	Chlorite (g)
0	0.033	0.1
1	0.066	0.2
2	0.066	0.2
3	0.066	0.2
4	0.066	0.2
5	0.066	0.2
6	0.132	0.4
7	0.132	0.4
7.5	0.132	0.4

After 8 h, the samples, which were fiberized, were removed from the solution, rinsed 10 times with deionized water, and placed in a covered glass Petri dish in a refrigerator.

### AAPH-treated sections

Three transverse 20-μm sections of spruce (1×1 cm) were floated in 30 mL of 10 mmol/L pH 4.5 acetate buffer and 20 mmol/L 2,2′-azobis (2-amidinopropane) dihydrochloride (AAPH) (Sigma Aldrich) at 29°C for 18 h. Under these conditions, we measured the AAPH half-life to be 436 h by following its absorbance using a UV/Vis spectrometer (Hatachi, U-3010), so 420 μmol of carbon-centered radicals are expected, and these rapidly add oxygen to give peroxyl radicals. After the reaction, the sections were thoroughly rinsed with DI water and placed in a covered glass Petri dish in a refrigerator.

### Fungus-exposed sections

Spruce blocks, as described above, were exposed to fungal hyphae by placing them on nylon mesh in a Petri dish containing potato dextrose agar that had been inoculated beforehand and fully colonized by individual fungi. All isolates were obtained from the Center for Forest Mycology Research, Forest Products Laboratory, Madison, WI, fungal culture bank. Incubations were conducted with the following isolated basidiomycetes at the indicated temperatures: *Postia placenta* MAD-698-R (29°C), *Phanerochaete chrysosporium* BKM-F-1767 (29°C), and *Serpula lacrymans* Harms-888-R (23°C). After exposure to the fungi for 3, 5, or 7 days, the blocks were placed in a parafilm-sealed glass dish and frozen to stop any further growth. The blocks were thawed, and transverse sections were cut from the interior of blocks using a microtome. The sections were obtained by cutting the blocks with a saw and then sectioning the cut surface. Relatively complete sections were obtained, but they were noticeably more fragile than fresh wood sections.

### Model substrates

SigmaCell type 101 cellulose (Sigma Chemical Company) was used as delivered. Enzyme-treated, milled spruce wood lignin was prepared by the method of Obst and Kirk [34]. Carboxy methylated agarose ion exchange beads (CM-beads), 50–200 mesh (Bio-Rad), were used after washing and centrifuging several times with distilled, DI water. Oxidized cellulose was prepared following the method of Floor *et al*. [35] using northern bleached softwood kraft pulp as the starting fiber.

### Staining procedure

Three sections were placed in a 100-mm Petri dish. Then 15 mL of 50 μmol/L AO solution in 100 mmol/L phosphate buffer, pH 7.1, was added. These samples were left in the dye solution, covered, and placed in the refrigerator for 24 h. For imaging, the sections were placed on slides with cover slips without washing. (Researchers are reminded that as a DNA-intercalating dye, AO has the potential to be mutagenic. The use of a low concentration dye solution reduces this risk, but care should be exercised to avoid direct human exposure to AO solutions.)

### Imaging

Confocal imaging was done on a Zeiss 510 Meta confocal laser scanning microscope, using a 1.2 NA 40× water objective. Images were acquired in 2-μm-thick optical sections and a pixel width of 0.45 μm. With 458-nm excitation, green emission was collected at 500–550 nm and red emission was collected with a 560 nm long pass filter.

Fluorescence lifetime imaging microscopy (FLIM) was performed on a Leica SP5 II scanning laser confocal with an integrated PicoQuant FLIM system (PicoQuant PicoHarp 300 TCSPC module) at the University of Pennsylvania Penn Vet Imaging Core Facility. FLIM excitation was at 470 nm (PicoQuant Sepia II multichannel picosecond diode laser) with 505–530-nm emission. To take the confocal images, the sections were excited at 488 nm with detection at 500–540 nm for green and 600–670 nm for red emission.

Widefield fluorescence images were taken with a Flea3 digital camera model FL3-U3-88S2C-C (Point Grey) mounted via C-mount adaptor on a Leitz Orthoplan epifluorescence microscope with 390–490-nm excitation and a 515-nm long pass filter, with a xenon-arc lamp. With each transverse section, 10 different spots were imaged, 5 earlywood and 5 latewood. Three different sections were imaged, for a total of 30 images per treatment. For the longitudinal sections, only 5 images were taken, and we did not try to isolate earlywood and latewood cells in these images.

Images were acquired using FlyCapture 2.6 (Point Grey). The camera was operated in manual mode, the gain was set to 0 db, with a gamma correction exponent of 1 (i.e., no gamma correction), and fixed color balance values of 550 (red) and 800 (blue). Shutter speed was adjusted to give maximum intensity values less than 255. Images were color-interpolated, using the “nearest neighbor” algorithm and stored as uncompressed TIF files. The interpolation is required because the camera sensor uses a Bayer tile pattern and obtains only a red, green or blue value at each pixel. The pixels are placed in groups of four (two green, one red, and one blue), and missing color intensities are obtained by copying values from other pixels in the group. To account for this over-representation of resolution, images using this camera system were downsampled by a factor of 2 in each dimension to better reflect the actual resolution.

Images were processed using Python 3.3 script. The image tools were largely based on the Python Image Processing Library Pillow 2.2.1. The script is available in the data archive. Briefly, images were first separated into three channels (red, green, and blue). A threshold mask was generated by choosing a lower limit value in the green channel that removed contributions from cells in the radial system and solution in cell lumina. The dark intensity value was determined by taking an image with a dust cover covering the camera, 12 for all three channels. This value was subtracted from all the data. Using the mask to select only cell wall regions, the red-green ratio was calculated pixel by pixel. Although the Bayer tile pattern of the camera sensor results in four approximately duplicate values for each pixel grouping, the calculated averages are still correct. Data were compiled as histograms and are presented as arithmetic averages for whole sets of images. Because the pixel color filters have some spectral overlap (for example, pure red emission will produce a weak signal in the green channel), the dynamic range for the ratios was rather limited. Typical values ranged from 0.5 to 2.5.

### Titration

Titration of anionic groups on various substrates was tracked by following solution conductivity. A house-built, computer-controlled system operated a pump and measured conductivity. An electromechanical diaphragm pump (Cole-Parmer, self-priming, micro pump) delivered titrant with a rate of 20 μL/pulse and 10 pulses/min. A typical titration required approximately 90 min. Data from a conductivity meter (Eutech Alpha Cond500) were acquired with a National Instruments USB-6001 board and software written in Python 3.3 with the pyDAQmx module. The sodium hydroxide used for titration was nominally 12.5 mmol/L and titrated against a known mass of dry potassium hydrogen phthalate. The sample mass was adjusted to give approximately 50 μmol of acid groups to titrate. For the wood, the titration was conducted with 100 sections. Data were analyzed using Microsoft Excel following the method of Scallan [36].

### Isothermal titration calorimetry

Isothermal titration calorimetry (ITC) measurements were performed using a VP-ITC calorimeter (MicroCal). The concentration of AO was 800 μmol/L in sodium borate buffer at pH 9. The CM-beads were added to the ITC cell to give a concentration of 50 μmol/L of carboxylic acid groups in sodium borate buffer. At this pH, the carboxylic acid groups should be deprotonated with sodium as the counter ion. Injections of AO into buffer and buffer into CM-beads were also conducted. Analysis of the ITC data was done with Microsoft Excel following the methods described by Grolier and del Rio. [37] As part of this effort, the significant heat contribution of the disassociation of the AO dimers upon dilution was removed from the data using a first principles model and a fixed equilibrium constant (3040 L/mol, ΔH = –44.4 kJ/mol) [22].

The total concentration of AO strongly adsorbed on the CM-beads was determined by washing beads in deionized water three times and then diluting in a known volume of 0.1 mol/L calcium chloride, which displaces the adsorbed AO with calcium ions. After filtration to remove the beads, solution absorbance was measured with a UV/Vis spectrometer (Hitachi, U-3010). The AO concentration was then calculated using an extinction coefficient of 33,016 at 467 nm, which was determined from a standard curve.

## Results and Discussion

### Staining of sound wood

**Fig 2:**
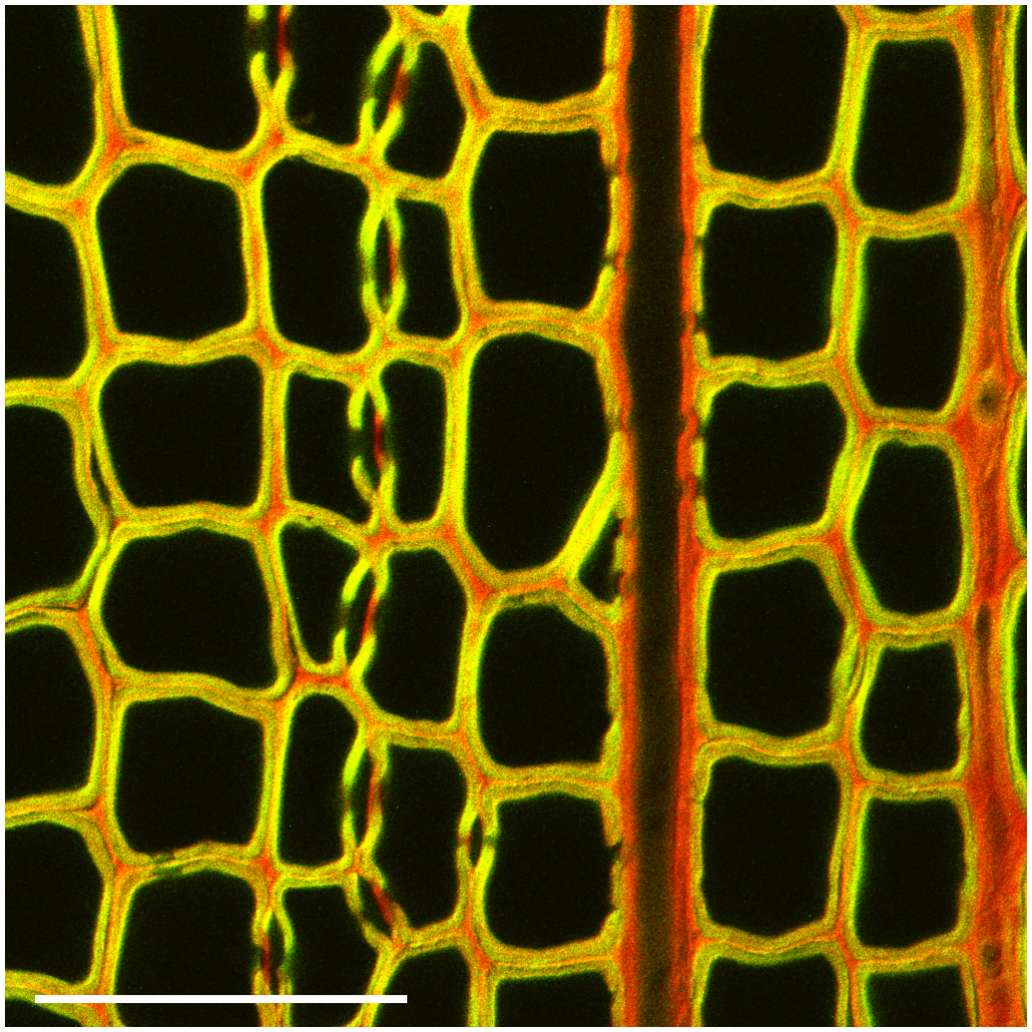
Combined red and green channel confocal images of AO-stained spruce early wood. The staining conditions were 50 μmol/L acridine orange in phosphate buffer, pH 7. The scale bar is 100 μm.

Our investigations of the mechanism for AO adsorption on wood required a rapid method that gives samples with a well-defined dye concentration history. Because this approach precluded the use of destaining procedures typical in classical microscopy (for an example of such an image see S1 Fig) we stained specimens using a relatively low dye concentration that was nevertheless sufficient for the formation of red-emitting AO dimers (50 μmol/L in phosphate buffer, pH 7.1) and then imaged them without further manipulation. S2 Fig, shows how the data change in response to exposure to other dye concentrations. A combined color image of a sound spruce AO-stained section obtained by laser-scanning confocal microscopy is shown in Fig 2. As previously observed, both red and green fluorescence occurred. Secondary cell walls emitted more green light. Close inspection of bordered pits shows that they appear greener than any other structure. Cell corners, middle lamellae, rays, and pit membranes emitted more red light. The fluorescence color pattern mostly correlates with the chemical composition of various wood cell wall regions. The mature secondary cell wall layers, S_1_, S_2_, and S_3_, are expected to be lignified with few charged polysaccharides exposed [38]. Although cell corners and middle lamellae have even higher proportions of lignin, they also contain pectic substances that are chemical remnants of the primary cell wall [38–40]. Pit membranes are known to have practically no lignin but significant quantities of pectins, especially in the margo [41, 42] Similar variations of color in confocal fluorescence microscopy have been observed when wood cells have been stained with safranin [43]. The colors in Fig 2 agree with the hypothesis that AO adsorbs as monomers on lignin in isolated sites, which results in green emission, whereas charged polysaccharides such as pectins provide sites that favor dimer formation, and thus red emission.

The one apparent contradiction is the orange color of the cell corners and middle lamellae. These regions are known to be higher in lignin than the secondary walls, so one might expect them to appear greener. To address this issue, we performed fluorescence lifetime experiments. This approach is based on the fact that the rate of energy loss depends on molecular level associations. When a molecule absorbs a photon of light and is pushed into an excited state, it can lose energy by emitting a photon (fluorescing), by dissipation as heat to the matrix, or by fluorescent resonance energy transfer (FRET) to another molecule. Thus the rate at which the fluorescence is lost is in part an indication of how electronically coupled a molecule is to its environment [44, 45]. Using pulsed laser excitation, one can use a confocal microscope to measure fluorescence lifetime, which is on the order of nanoseconds.

**Fig 3:**
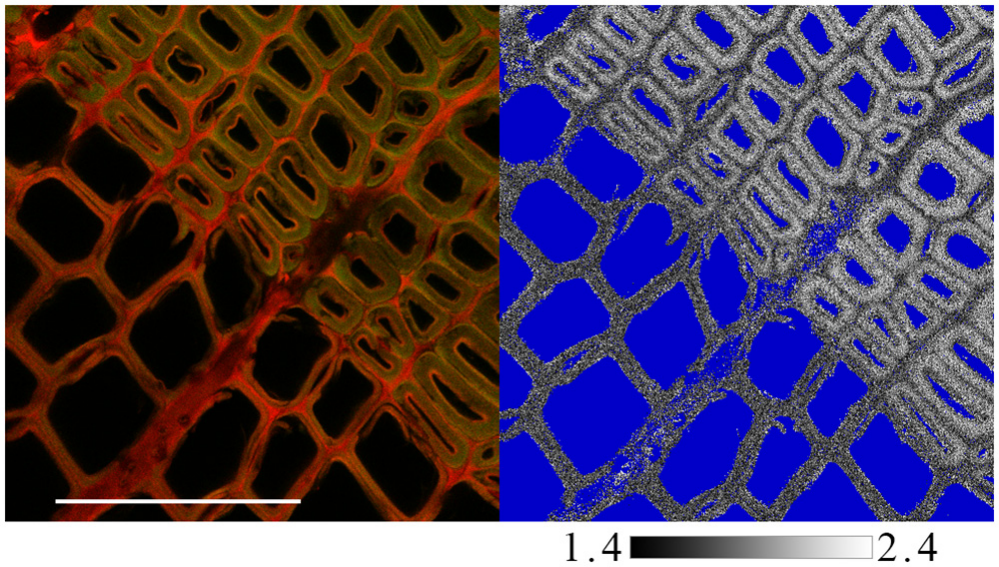
Images of confocal fluorescence (left) and lifetime for green fluorescence (right). The blue regions in the lifetime image were not analyzed. The scale bar is 100 μm.

We acquired confocal images and fluorescence lifetime data pixel by pixel on AO-stained wood sections. Fig 3, a side-by-side image of confocal and green emission lifetime measurements, shows that the secondary walls of the highly lignified latewood, which appear green in the confocal image, have relatively long lifetimes, 1.9 ns. By contrast, the middle lamellae, which is redder in the confocal image, has shorter green emission lifetimes, 1.6 ns. We interpret this result to mean that, although the middle lamella is highly lignified, the green emission in these regions is suppressed by FRET from green-emitting AO monomers to nearby red-emitting AO dimers. This interpretation is consistent with an earlier hypothesis that lignin in the middle lamella is intimately associated with pectins [38], whose pendant carboxyl groups are expected to stabilize AO dimers.

### Contributions of individual wood components to AO fluorescence

We used isolated wood cell wall components to confirm our observations that the colors in Fig 2 appeared to correlate with chemical composition. Although confocal microscopy provides good resolution and color channel separation, it requires specialized and expensive instrumentation. We switched to widefield fluorescence microscopy with computerized image analysis to quantify red-green ratios. S3 Fig shows monomer and dimer emission spectra overlaid on the spectral response curves for the three color channels of our camera. These data show that for this dye and our camera, dimer emission is responsible for most of the intensity captured by the red channel and monomer emission is responsible for most of the intensity captured by the green channel. Thus, red-green ratio is strongly correlated to dimer-monomer emission ratio.

**Fig 4:**
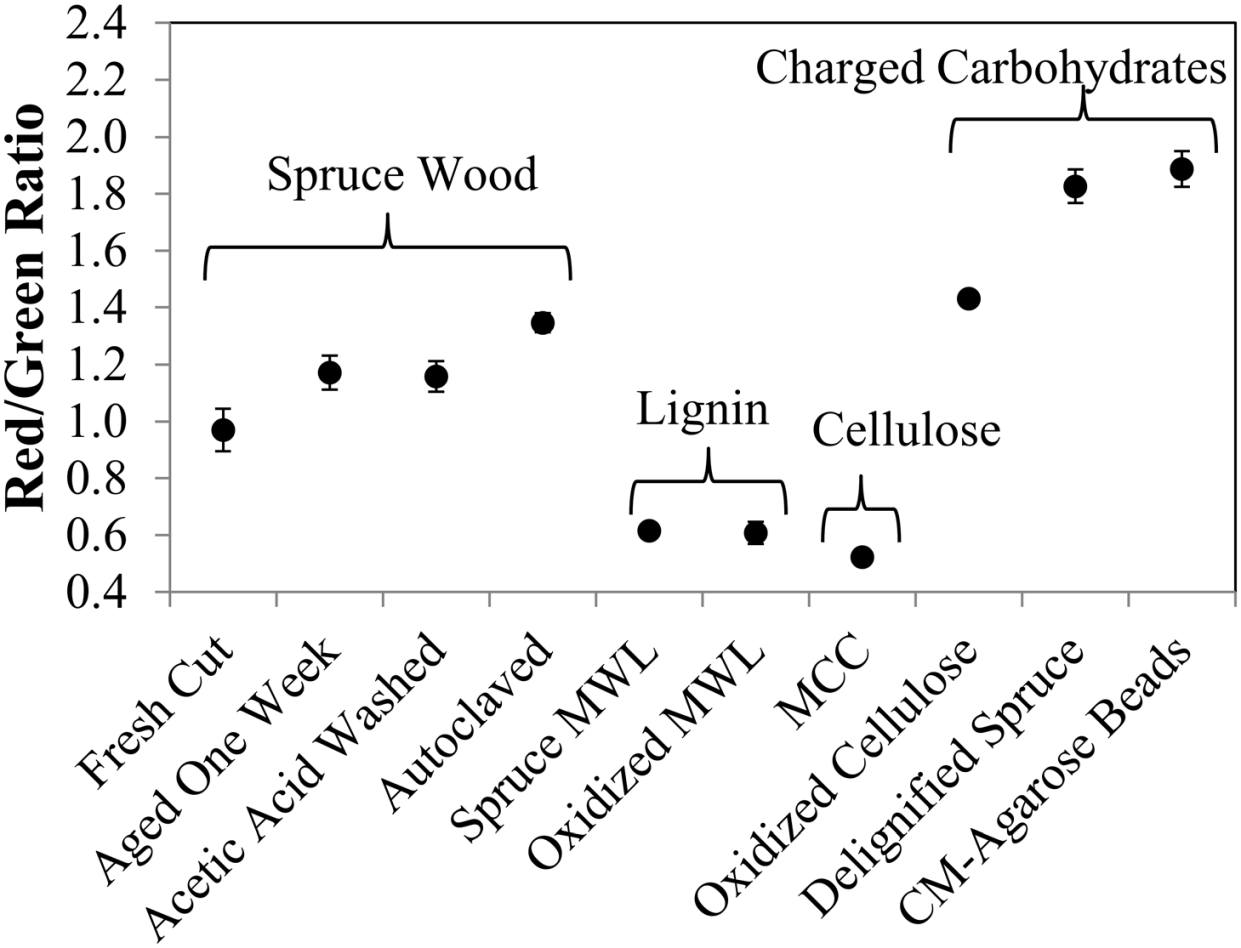
Red-green ratio calculated pixel by pixel from widefield fluorescence micrographs. Error bars represent 95% confidence intervals from analyzing images from five different locations in the earlywood on three different sections. MWL—milled wood lignin, MCC—microcrystalline cellulose, CM—carboxymethyl.

Red-green ratio data for various substrates are shown in Fig 4. Under our staining conditions, freshly sectioned spruce wood had an average red-green ratio near 1.0. If the wood was aged, acid-treated, or autoclaved, the ratio shifted slightly upward. Delignification of the wood by acid chlorite treatment, which leaves the hemicellulose and cellulose largely intact [33], resulted in a marked increase in the ratio to give a strong red emission.

Milled wood lignin (MWL) [34] exhibited a low red-green ratio, which was not changed by mild acid-chlorite oxidation. Thus, neither sound nor oxidized lignin is likely responsible for the increased red-green ratio seen in oxidized wood. As has been reported before, microcrystalline cellulose (MCC) adsorbed AO to give the green emission of monomers [30], but introduction of charged groups by periodate oxidation [35] resulted in a shift to more red emission.

Although using highly refined components (MWL and MCC) to represent the behavior of lignin and cellulose in wood is certainly an oversimplification, the fact that neither led to AO dimer formation under our staining conditions suggests that hemicelluloses or pectins are responsible for providing binding sites that lead to red emission. This conclusion is consistent with the observation that AO forms dimers in the presence of acidic polysaccharides [46]. For our particular staining conditions, there does not seem to be a simple linear relationship between acid group content and red-green ratio, as shown in Table 3, which indicates that the mere existence of an acid group is not a sufficient condition for dimer association. Other factors must be involved.

**Table 3:** Acid group content and red/green ratio.

Sample type	Acid groups μmol /g	95% CI	R-G ratio	95% CI
Microcrystalline cellulose	37	2	0.52	0.01
Fresh-cut spruce	79	4	0.97	0.07
Acetic-acid-washed spruce	117	6	1.16	0.05
Acid-chlorite-treated spruce	186	9	1.69	0.05
Fully delignified spruce	482	24	1.83	0.06
Oxidized cellulose	571	29	1.43	0.02
Carboxymethyl agarose beads	1118	56	1.89	0.06

95% CI = 95% confidence interval calculated using a Student’s *t* formalism

**Fig 5:**
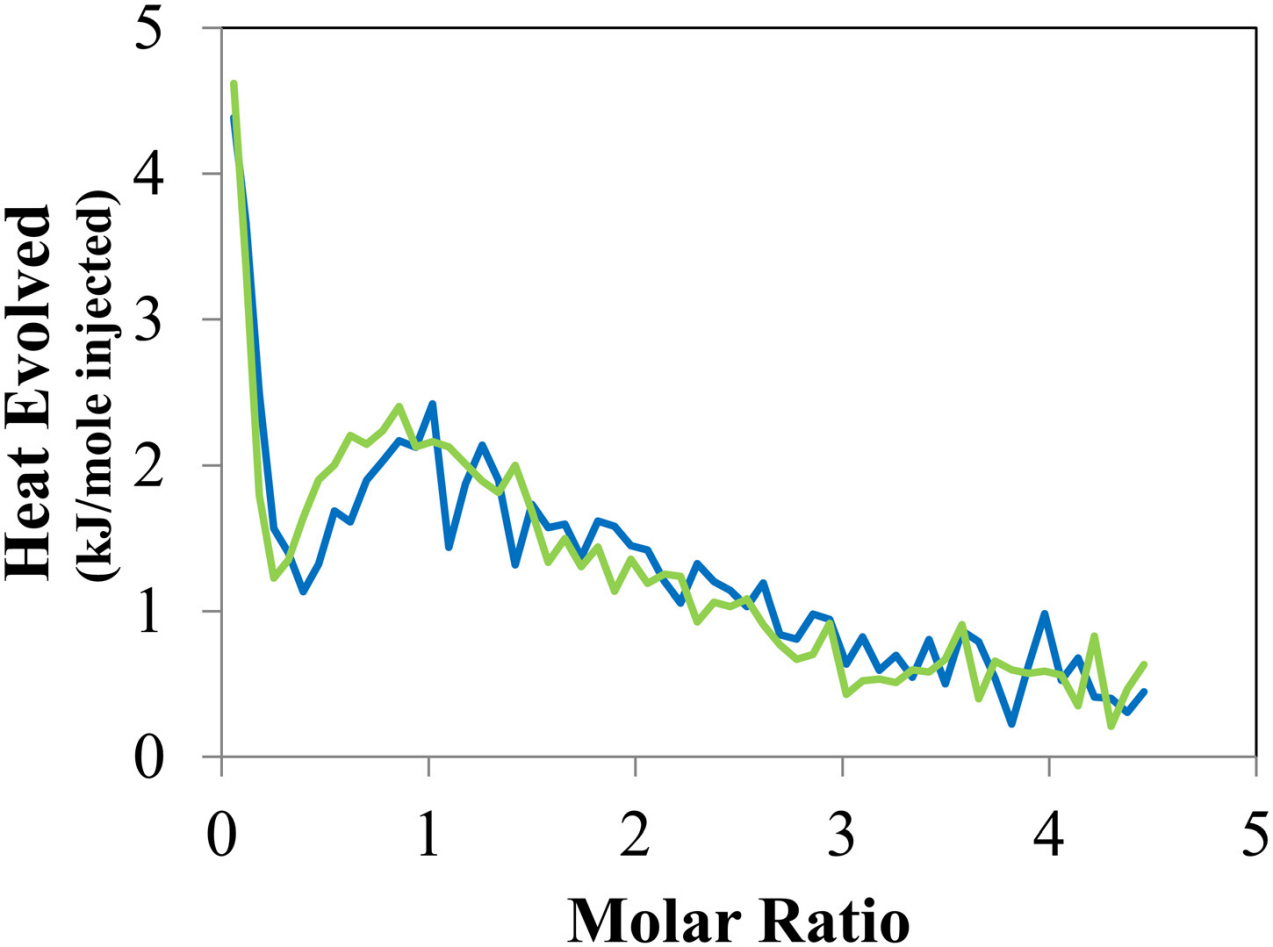
Replicates of ITC heat signature of acridine orange association with carboxymethylated agarose beads.

Because pectins and hemicelluloses are soluble in water, they are not amenable to our microscopic analysis methods. Instead, we used carboxymethylated agarose beads (CM-beads) to further explore the binding of AO to acidic polysaccharides. When CM-beads were stained using our standard conditions, they showed significant red emission. This water-swollen gel appears to provide a unique set of sites that allow AO molecules to associate with carboxyl groups while stacking with other AO molecules. Fig 5 shows the heat signature of AO adsorption on CM-beads obtained using isothermal titration calorimetry (ITC). Inspection of this figure shows strong exothermic binding between AO and a limited number of sites on the CM-beads during the first few injections. Consistent with this observation, titration of acid groups and dye adsorption studies on the same samples showed that 4.5 ± 1.5% (95% CI) of the acid sites participate in an interaction that results in this strong association. After the strong interaction during the first few injections, we observed a slow heat evolution consistent with simple ion exchange. When these observations are combined, one is led to the conclusion that the existence of a single carboxyl group is not sufficient to facilitate dimer formation. Instead, a small number of sites in the CM-beads provide the correct environment for dimer formation. Extending the conclusions about AO dimer structure in water [23] to our observations with CM-beads suggests that two carboxyl groups in the correct orientation could each associate with an AO molecule and also allow an anti-parallel association between the two AO molecules, see Fig 1, which allows them to form the combined electronic state of a dimer. A similar structure has been proposed to explain RNA/AO association complexes [21]. Accordingly, the red emission exhibited by AO-stained wood reflects the presence of hemicellulose or pectin carboxyl groups that are sufficiently near each other to form ensembles that favor the adsorption of AO dimers.

We next considered the adsorbed species that produces the green emission. Because exposure of cellulose during fungal decay occurs only after significant lignin removal, as indicated by the Astra-blue staining results of Srebotnik and Messner [2], it is unlikely that cellulose surfaces provided many AO adsorption sites in our sound wood sections. Instead, lignin is likely the wood cell wall component that contains the adsorption sites for AO that result in green emission. The same conclusion was previously reached by Stockert *et al*. [27] and confirmed by Bond *et al*. for safranin [43]. We also noted above that MWL stained with AO gave a strong green emission. Because AO molecules are cations under our buffer conditions and if no carboxylic acid groups are available to balance the charge, there will be a tendency of AO molecules to repel each other and associate at isolated sites.

### Staining of lightly oxidized wood

**Fig 6:**
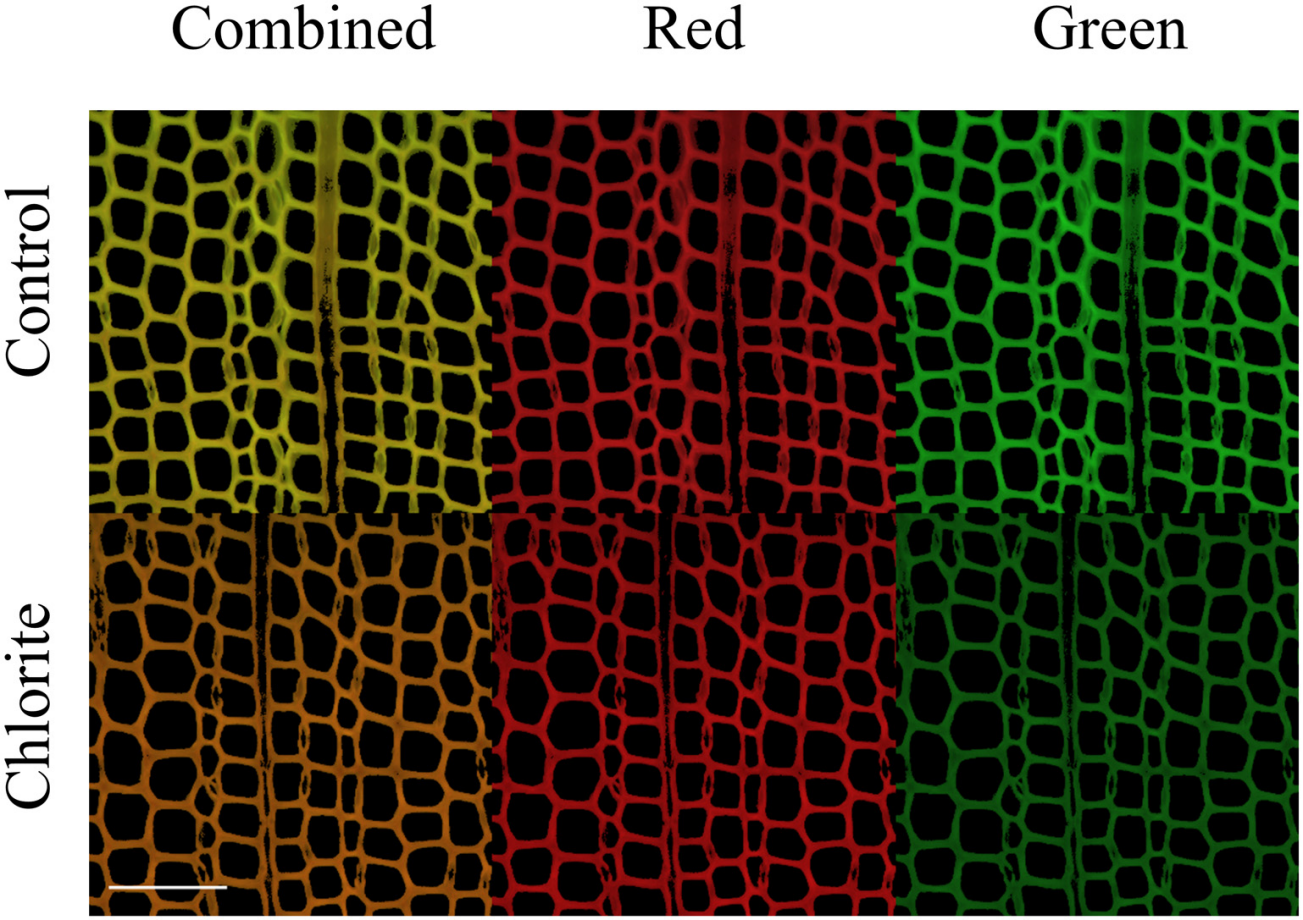
Fluorescence micrographs of control and chlorite-oxidized spruce wood sections. The images show that green intensity decreases upon treatment. The scale bar is 100 μm.

The impact of oxidation on AO staining of wood was assessed by exposing spruce sections to a small amount of acid chlorite or AAPH. The amount of chlorite added for this treatment (1 mmol/g oven-dried wood) was approximately 1% of the amount required to fully delignify the same wood. After this treatment, wood sections remained physically intact. Comparing the masses of 100 control and 100 chlorite-treated sections showed that less than 5% of the wood biopolymers were removed during this treatment. Fig 6 shows widefield fluorescence micrographs of control and chlorite-treated sections. Note that the fine detail typical of a confocal image is lost in such images due to excitation of out-of-focus emitters. Inspection of Fig 6 shows that the apparent color shifted away from green and towards red after light oxidation of the wood.

The observed shift could arise from either an increase in red emission or a decrease in green emission. To resolve this question, we separated the combined images into their corresponding red and green color images, which are also shown in Fig 6. When one compares the two red images, it is evident that oxidation did not cause apparent intensity to change appreciably. By contrast, there was a pronounced reduction in the green intensity of the image for the chlorite-treated section. To quantify this change, we made histograms of pixel intensity and calculated modes for 10 treatment images and 10 control images. While the red maximum intensity decreased by 12% (75 for control and 66 for chlorite-treated), the green maximum intensity showed a greater decrease of 34% (65 for control and 43 for chlorite-treated).

Although some chemical reactions that occur between acid chlorite and lignin are similar to those proposed for fungal systems, e.g., outer sphere electron transfers, others are not. AAPH under aerobic conditions is known to generate oxygen-centered radicals that react with lignin model compounds to give product distributions that are similar to those produced by the fungal enzyme lignin peroxidase [47]. We treated wood sections with AAPH for 18 h under conditions that are expected to yield 1.4 mmol/L of oxygen-centered radicals. After this treatment, the red-green ratio of AO-stained sections was 1.67 ± 0.03, which can be compared to 1.69 ± 0.05 for the acid-chlorite-treated wood and to 0.97 ± 0.07 for the fresh-cut wood.

In summary, the change in color illustrated in the combined color image is due to a loss in green intensity and not to an increase in red intensity. This result supports the hypothesis that the increased red-green ratio obtained upon AO staining of oxidized wood is attributable to a loss of certain exposed lignin structures that bind green-emitting AO monomers rather than to a gain in the number or accessibility of acidic structures that bind red-emitting AO dimers. The reactions leading to reduced AO adsorption on lignin can be caused by both acid-chlorite and the biomimetic oxidant AAPH.

### Response to fungal colonization

**Fig 7:**
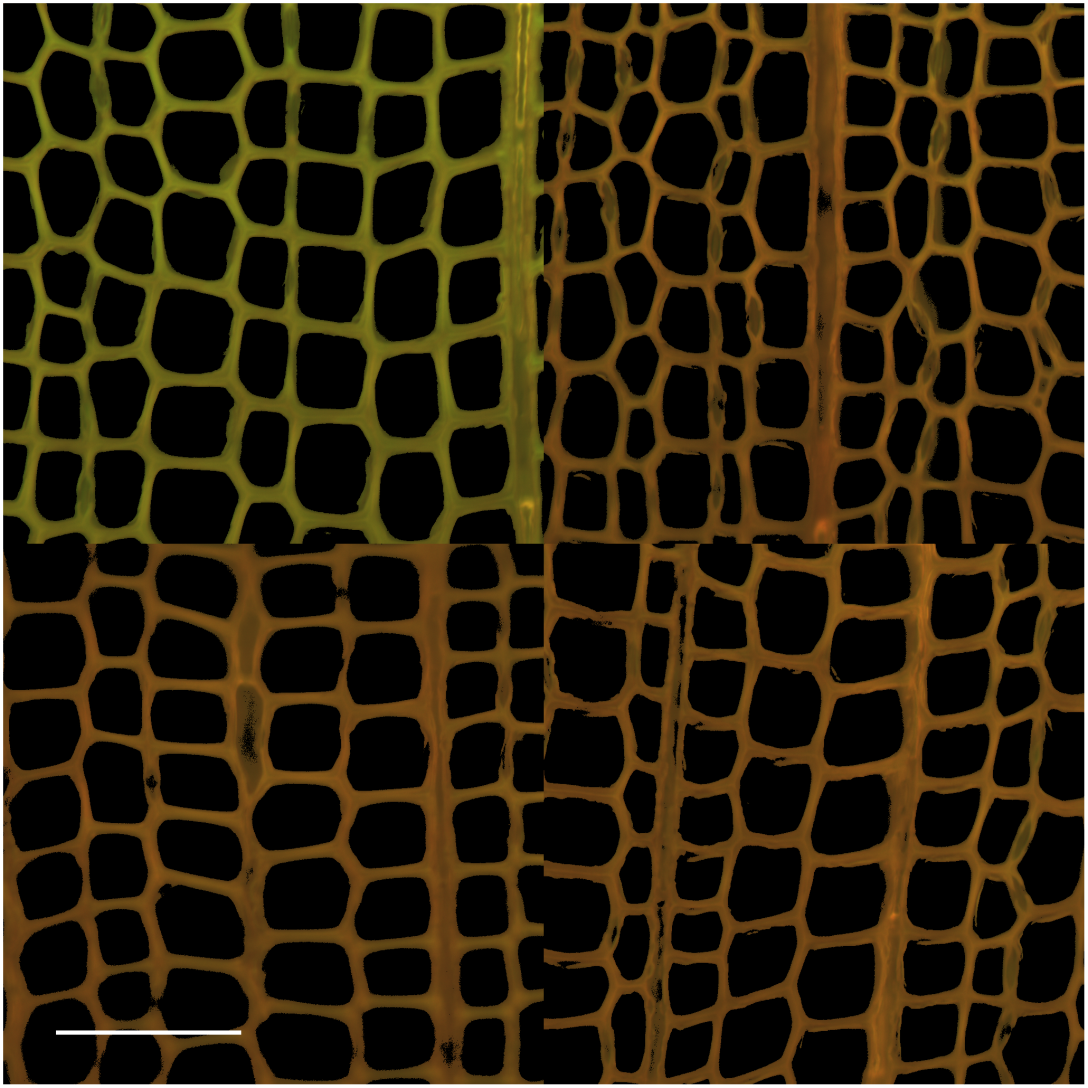
Fluorescence micrographs of AO stained samples. Control wood (upper left), after 7- day colonization by *Postia placenta* (upper right), *Phanerochaete chrysosporium*, (lower left) and *Serpula lacrymans* (lower right). The scale bar is 100 μm.

As has been observed before [13], fungal exposure caused AO-stained wood sections to appear redder in fluorescence. Fig 7 shows a composite of fluorescence micrographs of control wood and sections of blocks colonized by three different basidiomycetes, the brown rot fungi *Postia placenta* and *Serpula lacrymans*, and the white-rot fungus *Phanerochaete chrysosporium*. In all three cases we observed an increased red-green ratio of fluorescence after AO staining. An important aspect of this result is that the decay mechanisms for brown rot and white rot differ considerably. Both types of fungi oxidize lignin in wood, but brown rot fungi employ a nonenzymatic Fenton system that generates biodegradative reactive oxygen species, whereas white rot fungus employs ligninolytic enzymes that include specialized peroxidases [5]. Thus, the AO staining results we obtained after fungal treatment point to a general oxidation of the substrate rather than to selective action by a particular oxidant.

Our data exclude an alternative hypothesis, that the observed shift in color after fungal treatment is related to a change in autofluorescence. To test for this possibility, we acquired images for the same wood samples without AO staining. Control spruce sections have green autofluorescence under our conditions, which is indistinguishable from the green emission from AO but with an intensity of only 4.0% ± 0.7% of the intensity of similar AO-stained sections. Colonization by each of the three fungi reduced autofluorescence to less than 0.13% ± 0.01%. Thus, no more than 4% of the color changes apparent in Fig 7 can be attributed to a loss of green autofluorescence.

**Fig 8:**
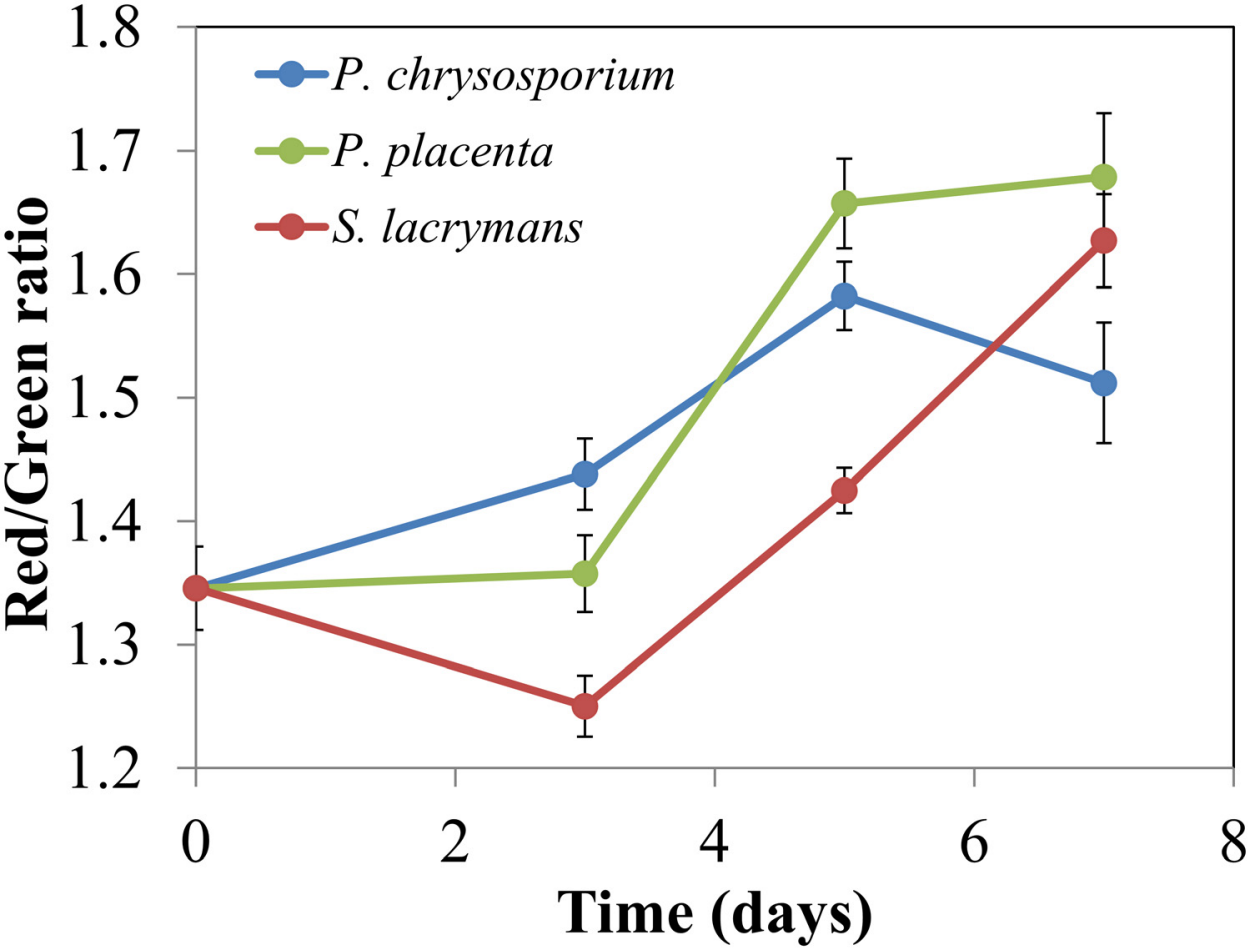
Red-green ratio calculated pixel-by-pixel from fluorescence micrographs. Error bars represent 95% confidence intervals from analyzing images from five different locations in the early wood on three different sections.

Color shifts in Fig 7 can be quantified by calculating the pixel-by-pixel red-green ratio for 15 different images and averaging them together. Fig 8 shows a plot of average red-green ratios for samples harvested at 3, 5, and 7 days after placement of wood blocks over nutrient agar colonized with each of the three fungi. Because the blocks were autoclaved before exposure to fungi, an autoclaved block was sectioned, stained, imaged, and designated as the 0-day sample. Although the 3-day data were similar to the 0-day value, the 5- and 7-day data clearly show that fungal colonization caused a reduction in green emission similar to that caused by a low concentration of acid chlorite or AAPH. Complete reduction of chlorine dioxide to chloride and water requires five electron equivalents per mole. Because the AO staining effect for the fungal treatments was similar to the low chlorite treatment, the effect of fungal action must be similar to transferring 5 mmol electrons/g of dry wood.

**Fig 9:**
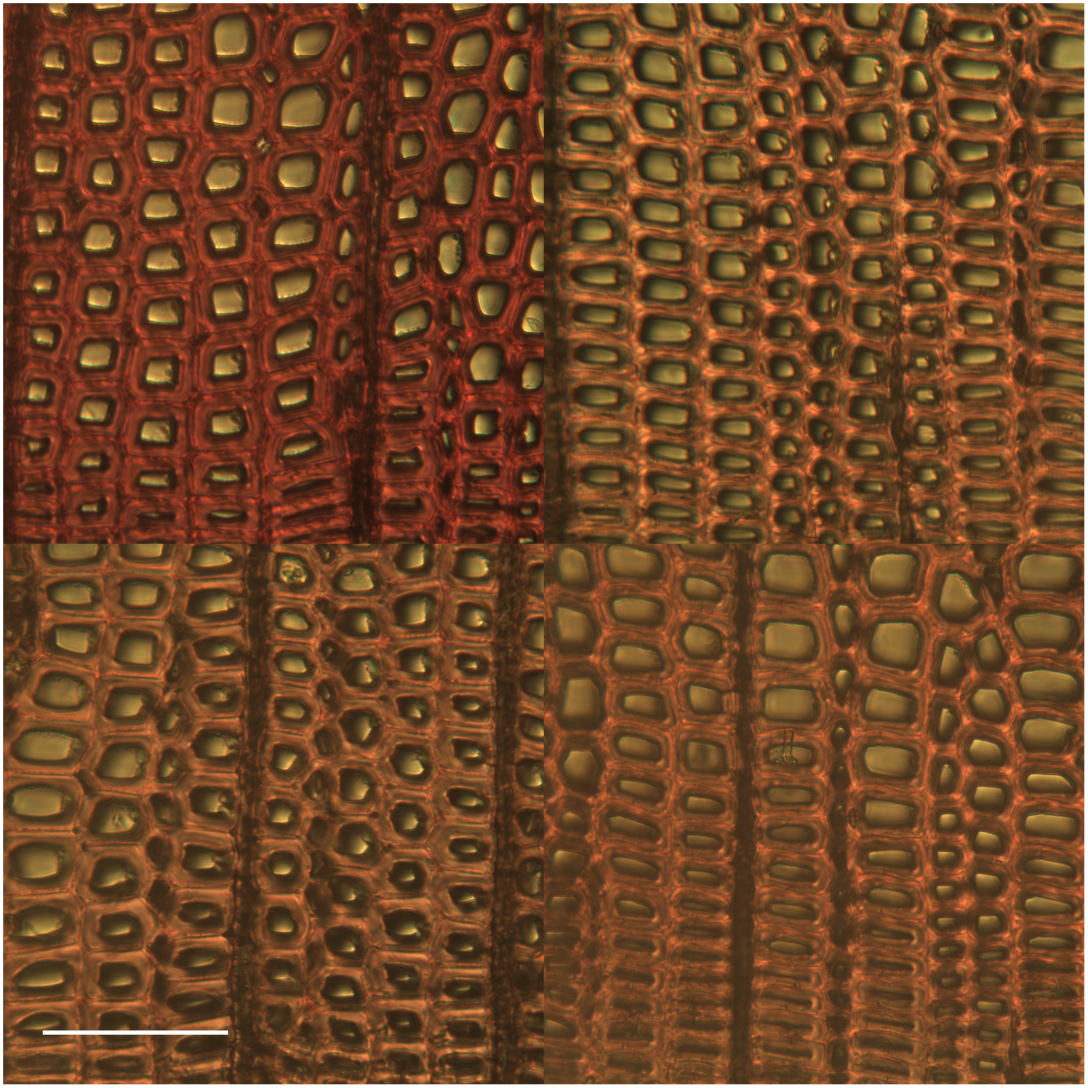
Transmitted light micrographs of phloroglucinol stained samples. Control wood (upper left), after seven day colonization by *Postia placenta* (upper right), *Phanerochaete chrysosporium* (lower left), and *Serpula lacrymans* (lower right). The scale bar is 100 μm.

Finally, the conclusion that fungal oxidation is a cause of reduced association of AO with lignin is consistent with results we obtained using phloroglucinol staining and transmitted light microscopy. The basis for this procedure is that phloroglucinol reacts with aryl aldehyde residues that occur naturally in native lignin, yielding reddish pigments [12]. Fig 9 shows a composite figure that can be compared with Fig 7. The significant decline in phluoroglucinol staining after colonization shows that fungi flooded the wood with sufficient oxidant to reduce the aryl aldehyde concentration. A likely explanation for this result is that aryl aldehydes are inherently susceptible to autoxidation [48] and consequently are unlikely to survive fungal attack. Although a reduction in red color is apparent in these images, it is difficult to perform quantitative analysis due to significant sample-to-sample variations in color intensity. The AO staining method, because it is based on ratiometric measurements of red and green emission, affords data that are less sensitive to such variations in intensity.

### Hypothesis confirmed

Our working hypothesis is that AO binds to lignin as isolated molecules, exhibiting green emission. Anionic polysaccharides such as pectins and hemicelluloses provide a different type of binding site, which likely has at least two carboxylic acid groups. These anionic sites provide a charge balance to allow an ensemble of two cationic AO molecules to associate with each other and form a combined electronic state that exhibits red emission. Considering the results of previous studies with AO together with our new results, our hypothesis appears to be supported.

### Implications for fungal mechanism

The data show that modifying lignin by oxidation reduces its association with AO. A rather small amount of oxidation, 5 mmol/g of electron equivalents, reduces the green intensity by 30–40%. Because the red emission remains relatively constant, this loss in green intensity results in wood sections changing color from green to orange when observed under a fluorescence microscope with blue excitation. Although it may seem surprising that a small amount of oxidation can cause such a strong reduction in adsorption, it is possible that oxidation is restricted to the water-accessible surfaces of lignin, which are where the AO molecules also bind. The specific chemical nature of the changes to lignin that cause reduced association with AO remains a question that must be addressed in future work.

Our results further show that colonization of wood by three wood decay basidiomycetes caused color changes in AO fluorescence that are consistent with incipient oxidation of the wood. Although fungal hyphae are not visible in the fluorescence images, we observed them in transmitted light micrographs. In 5 to 7 days we saw changes in AO staining, reduced phloroglucinol response, and loss of autofluorescence, at which time only a few percent of the lumina had hyphae in them. How so few hyphae can so uniformly modify wood on the scale of several hundred micrometers is an open question. What we can conclude is that the proximal oxidant must be of relatively low oxidation potential and low molecular weight. A low oxidation potential is required because a high potential oxidant would react with all adjacent lignin substructures, and consequently the oxidation would be limited to lumina containing hyphae. A low molecular weight is required because the pores in sound wood are too small to permit the transport of large molecules such as enzymes [49]. Accordingly, the proximal oxidant must be of low enough potential not to oxidize most lignin structures, yet must be able to react with aryl aldehydes, as indicated by the loss in phloroglucinol staining.

## Conclusions

Although wood sections stained with AO fluoresce with a wide range of colors, depending on treatment history, we can explain the observations by noting that lignin can adsorb AO molecules in isolated sites, which gives green emission, and anionic groups on carbohydrates can provide unique binding sites that promote AO dimer binding, which gives red fluorescence.

A small amount of oxidation is sufficient to greatly reduce the association of AO with lignin but leaves dimer association largely unchanged. Thus, wood sections under fluorescent microscopes change from green to yellow to red as oxidation progresses.

Finally, the brown and white-rot fungi in this study appear to be flooding wood blocks with relatively low oxidation potential and low molecular weight oxidants during early colonization. Oxidation reduces AO association with lignin, removes aryl aldehydes, and reduces autofluorescence. This AO-staining method allows researchers to track the progress of early fungal colonization and may facilitate identification of the biochemical processes responsible for modifications of wood biopolymers.

## Supporting Information

S1 Fig Widefield fluorescence micrographs of AO stained spruce wood sections, control (left), 3 days (middle) and 7 days (right) after inoculation with *P*. *chrysosporium*. The scale bar is 100 μm.
(PDF)

S2 Fig Red/green ratio for AO stained sections at various concentrations. Spruce blocks were colonized by three different fungi for seven days, then sectioned and stained with AO. Since the blocks were autoclaved before inoculation with fungi, an autoclaved block is used as a control.
(PDF)

S3 Fig Intensity versus wavelength for AO monomer and dimer and the camera quantum efficiency curves. The monomer and dimer are represented by control and chlorite-treated spruce sections. The quantum efficiency curves were adapted from the camera specification published by Point Grey [S1].
(PDF)
