# Supplementary material for: Acridine Orange Indicates Early Oxidation of Wood Cell Walls by Fungi
Source: PLoS One. 2016 Jul 25;11(7):e0159715. doi: 10.1371/journal.pone.0159715 (PMC4959780; doi:10.1371/journal.pone.0159715)
Supplement: S1 Fig — The scale bar is 100 μm. (PDF) [file pone.0159715.s001.pdf]

Supplement information for “Acridine Orange indicates early oxidation of wood cell walls by fungi”

AO staining using traditional methods

The observations that launched our study are shown in Fig S1. By using a high concentration of AO followed by exhaustive destaining, we obtained high-contrast images of sound and oxidized wood. Unmodified wood stained with AO had a strong green emission. Attack by the white rot fungus *P. chrysosporium* caused the emission to change progressively from green to yellow to red, and earlywood changes sooner than latewood. These results show that high concentration dye exposure followed by washing results in high contrast AO-stained wood sections, which are potentially useful for qualitative work because they illustrate modifications clearly.

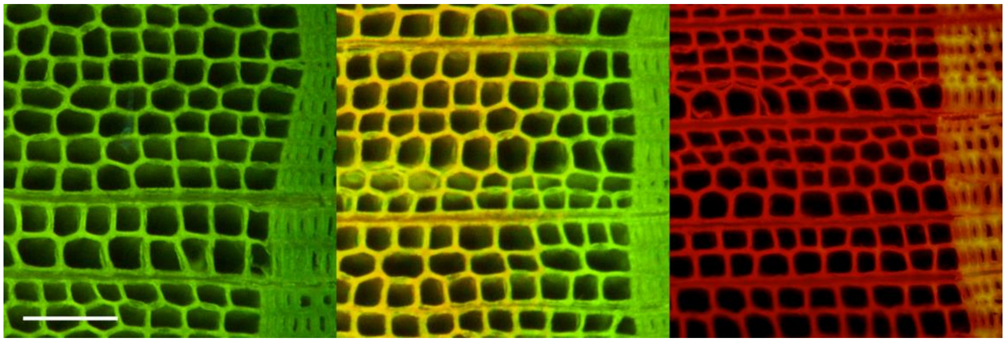

Fig S1: Widefield fluorescence micrographs of AO stained spruce wood sections, control (left), 3 days (middle) and 7 days (right) after inoculation with *P. chrysosporium*. The scale bar is 100 µm.

Supplemental Materials and Methods

High Concentration Dyeing and Washing

Sections were immersed in 0.01% AO at pH 7 for 5 min, and cleared in a series of ethanol-water mixtures (25%, 50%, 70%, 95%), for 1 hour in each concentration with several washes. Then the sections were brought back to water through a series of ethanol-water mixtures (70%, 50%, 25%) for 30 min in each concentration with several washes.
