# Supplementary material for: Acridine Orange Indicates Early Oxidation of Wood Cell Walls by Fungi
Source: PLoS One. 2016 Jul 25;11(7):e0159715. doi: 10.1371/journal.pone.0159715 (PMC4959780; doi:10.1371/journal.pone.0159715)
Supplement: S2 Fig — Spruce blocks were colonized by three different fungi for seven days, then sectioned and stained with AO. Since the blocks were autoclaved before inoculation with fungi, an autoclaved block is used as a control. (PDF) [file pone.0159715.s002.pdf]

Supplement information for "Acridine Orange indicates early oxidation of wood cell walls by fungi"

Selection of AO concentration for staining

Our investigations of the mechanism for AO adsorption on wood required a more rapid method that gives samples with a well-defined dye concentration history, which precluded the use of destaining procedures. We chose to use a solution of known AO concentration in buffer. Fig S2 shows the red-green ratio for various stained wood sections from wood that has been colonized with three fungi, plus the control versus AO concentration. As expected as that concentration of AO is increased, more dimers are formed and the sections emit more red light. In all cases, the fungus-treated samples have statistically significantly higher red/green ratios than control. We selected a value of 50 µmol/L.

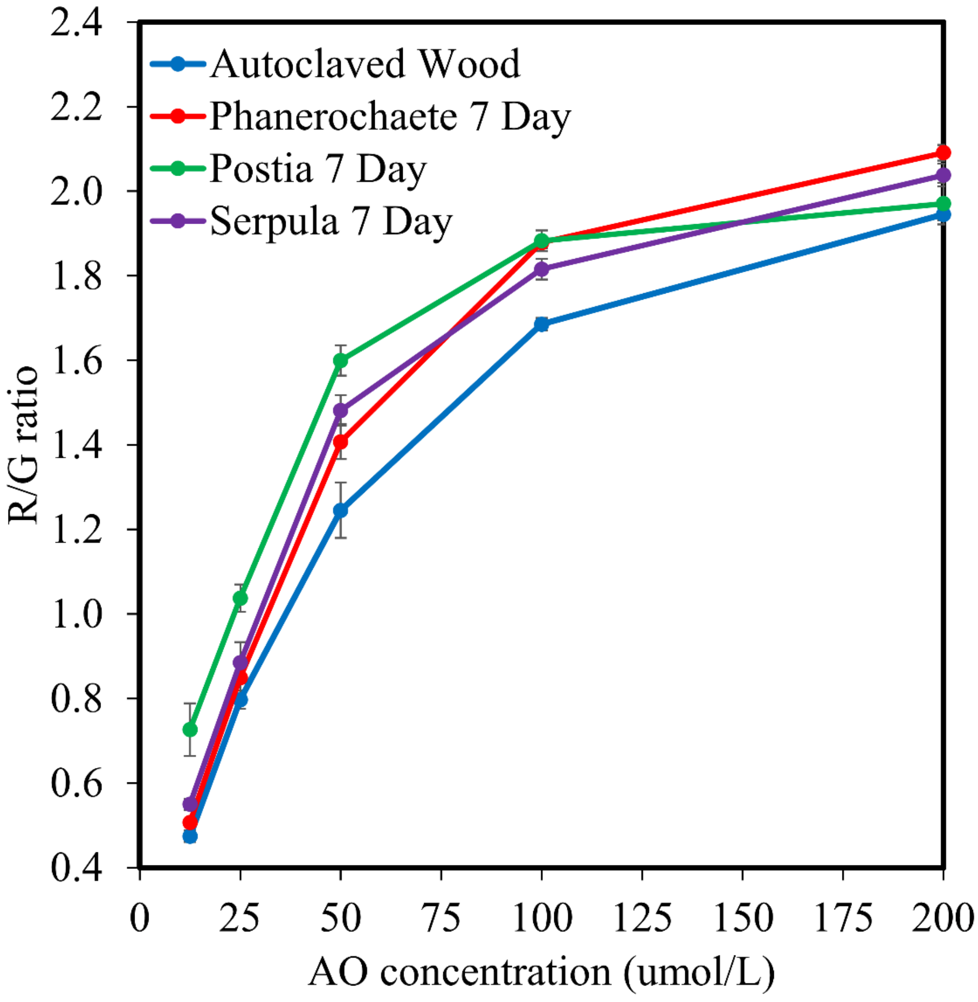

Fig S2: Red/green ratio for AO stained sections. Spruce blocks were colonized by three different fungi for seven days, then sectioned and stained with AO. Since the blocks were autoclaved before inoculation with fungi, an autoclaved block is used as a control.
