# Supplementary material for: Acridine Orange Indicates Early Oxidation of Wood Cell Walls by Fungi
Source: PLoS One. 2016 Jul 25;11(7):e0159715. doi: 10.1371/journal.pone.0159715 (PMC4959780; doi:10.1371/journal.pone.0159715)
Supplement: S3 Fig — The monomer and dimer are represented by control and chlorite-treated spruce sections. The quantum efficiency curves were adapted from the camera specification published by Point Grey [S1]. (PDF) [file pone.0159715.s003.pdf]

Supplement information for “Acridine Orange indicates early oxidation of wood cell walls by fungi”

Considerations on using a color camera to acquire images for quantitative analysis.

The human eye and brain translates incident light into the perception of an image through a rather complex process. Most digital camera systems attempt to correct the raw numerical data generated by an image sensor to best reflect what a human would perceive. These transformations introduce significant difficulties in using a camera as a scientific instrument.

Gamma correction is often applied to image data in an attempt to account for the fact the humans tend to perceive small differences in dark colors better than differences in light colors. Thus, the numerical value assigned to a particular pixel is often not linear with luminosity. In the particular case of this work, we would like to have the numerical values linearly related to emission intensity, so we set the camera to do no gamma correction, which corresponds to a gamma exponent of 1.

In addition, it is common for color cameras to perform other transformations to the image data. Often these transformations are done on the chip and cannot be turned off. The camera selected for this work was chosen because we could turn off these transformations and access the raw sensor data.

Another consideration was the alignment of the fluorescence emission spectra to the spectral response of the camera. Often researchers choose to use a monochrome camera and put color filters in the light path to select for particular wavelengths of light. This method allows for the

selection of filters to meet the needs of a particular experiment. If the filters on a color camera happen to align with the requirements of the experiment, a color camera can be used.

In color cameras a patterned color mask, also known as a Bayer filter, is overlaid on the light sensors, which are identical to those sensors in a monochrome camera. This mask modifies the spectral response of each pixel. Our particular camera has patterned color filter that provides red, green and blue pixels with an IR filter overlay that reduces light with a wavelength longer than 700 nm. Fig S3 shows the spectral response of the camera used for this work, as adapted from the data provided by Point Grey, the camera manufacturer [S1]. Note that the three channels do have some overlap, which results in limiting the range of R-G ratios. Also shown in Fig S3 are the emission spectra of a control (largely monomer) and chlorite-treated (largely dimer) sections stained with AO using the high concentration dye and washing method described in the supplemental materials and methods. Inspection of Fig S3 shows that the emission spectra are well-aligned with the spectral response of the green and red channels of the particular camera used for this work, and thus most of the intensity found in the red channel is due to the dimer emission and the intensity in the green channel is mostly due to monomer emission. Thus, R-G ratios measured with this camera should be linearly related to the ratios of emission intensities from AO monomers and dimers.

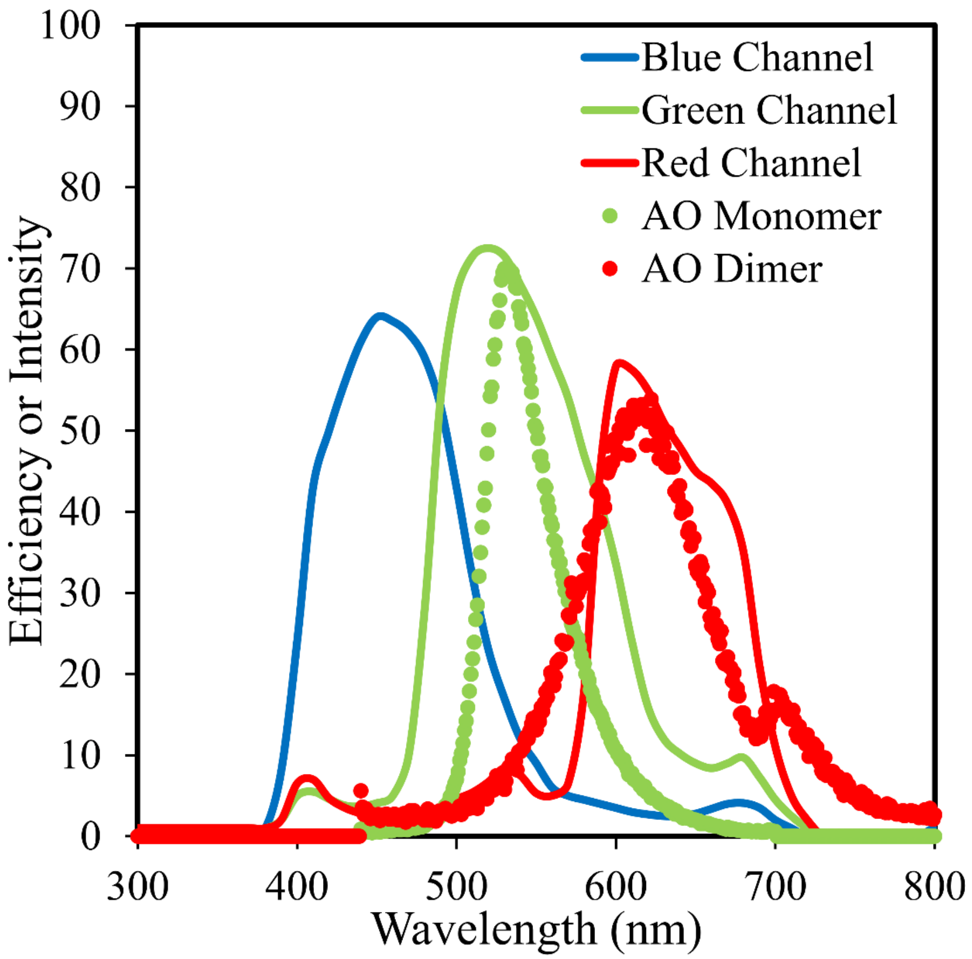

Fig S3: Intensity versus wavelength for AO monomer and dimer and the camera quantum efficiency curves. The monomer and dimer are represented by control and chlorite-treated spruce sections. The quantum efficiency curves were adapted from the camera specification published by Point Grey [S1], and used with their permission.

Supplemental Materials and Methods

High Concentration Dyeing and Washing

Spruce sections for Fig S3 were immersed in 0.01% AO at pH 7 for 5 min, and cleared in a series of ethanol-water mixtures (25%, 50%, 70%, 95%), for 1 hour in each concentration with several washes. Then the sections were brought back to water through a series of ethanol-water mixtures (70%, 50%, 25%) for 30 min in each concentration with several washes.

Emission spectra of wood sections

Front face fluorescence emission was acquired from AO-stained transverse sections of white spruce (1×1cm) in the solid sample holder of a Fluorolog Tau-2 fluorescence spectrometer (SPEX inc.). Emission was scanned with excitation set at 420 nm. Both excitation and emission monochrometer slits were set to 5 nm bandpass.

Supplement references

S1. https://www.ptgrey.com/support/downloads/10110
